# The Presidency of the American Medical Association

**Published:** 1856-11

**Authors:** 


*The Presidency of the American Medical Association.*—Our proposition to preserve the dignity of this office, and to make it in some sense an expression of the voice of the profession on the merits of its distinguished men, is readily adopted by independent journalists. The following high-toned and generous article is from the Nashville Medical Journal. It is the more noteworthy, from the fact that the next meeting of the association is to be held at Nashville:

"We endorse without hesitation the views of the Buffalo Journal. We regard the position of President of the American Medical Association as far more exalted than that of Speaker of the United States House of Representatives, and think that the members of our Professional Congress prove untrue to themselves and the great body of learned and scientific men they represent, whenever they place themselves in a condition to invite suspicion that their selection of a presiding officer was influenced by the members from the State in which the meeting was held. The fact that we happen to be editor of a medical journal at the point selected by the Medical Association as their place of meeting next year, gives us no authority to speak for others, here or elsewhere, nor upon the other hand, should it prevent us as an individual physician from speaking for ourself, especially when the subject is undergoing discussion in the journals. Our opinion is, that in selecting this officer we should have an eye single to the professional and moral character of the individual without attaching the slightest importance to his place of residence. He should be a "working-man," and especially should he have toiled *in* and *for* the association. We believe that the nomination of this officer should be taken from the nominating committee, and that he should be elected by the association by ballot. In no other way can the presiding officer be regarded as the representative of the association. In this way the promotion of a member to this exalted office would be to confer upon him the highest honor in the power of his professional countrymen. This plan would secure the attendance of the most distinguished of our brethren, for the selection would necessarily be made from those present. But when it is understood beforehand that this office is rather the result of the accidental conjunction of the place of meeting and place of residence, the position cannot be regarded as a distinguished honor. Every physician who loves his profession must be ambitious of the good opinion of the more high-minded and honorable of his brethren, and cannot be indifferent to such honors as they can through their public meetings and associations confer upon him, and to this extent every one may be regarded as feeling more or less personally interested in the presidency of our association, the very highest position which our suffrage can secure to the worthy among us. The framers of our constitution could not have foreseen the influence of local circumstances in determining the presidency of the association, but as experience clearly shows that such is the fact, it is apparant that it is now the first great duty of this body to so amend the constitution as to secure a more just and equitable method of appointing its president. But as the present provisions cannot be repealed at the next meeting, a constitutional provision requiring that resolutions to change that instrument shall lay over to the next meeting, our brethren abroad may very naturally expect in advance some expression of the feelings of the profession in this State upon that point. Those with whom we have discussed this subject here agree that the *habit* of the association in selecting its presiding officer from the city in which the meeting occurs, is a bad one, and cannot fail to destroy the dignity and honor of the position, and consequently impair the honor, dignity and usefulness of the association, and that the sooner a bad habit is corrected the better, and do therefore most earnestly hope that the reformation may begin at Nashville at the next meeting of the association.

"Our own preference for the next president is a "working man" in a neighboring State."

The Medical Independent, of Detroit, also seconds the proposition in the following remarks. For the complimentary expressions toward ourselves we can only tender our thanks, and assure the Independent of a reciprocity of sentiment:

"*An Evil Exposed, and its Remedy Proposed.*—Dr. Sanford B. Hunt has presented in his leading editorial for September, some very *pointed* and forcible remarks upon '*The Presidency of the American Medical Association.*' The independence and ability which characterize his pen in laboring to correct existing evils in the profession, as well as in the development and promulgation of scientific truth, has given to the Buffalo Medical Journal a commanding position in our medico-periodical literature.

"We fully appreciate the comments which he has made upon the 'machinery' which manufactures presidents for the association, and confidently believe they will be endorsed by every member of that body, who values the highest interests of the organization above clique subserviency and personal notoriety.

"There is no doubt that the degrading tendency of the *evil* has done much to impair the respectability of the association, and therefore to paralyze, to no inconsiderable extent, its elevating and progressive influence upon the profession. It has stigmatized that body with the character and reputation for making *great* men out of very *small* material. Now this is carrying *mechanical improvements* a little too far. Let such facilities belong to the secular press, and the 'pathies' and 'isms' of the day; while to the association belongs the object for which it was organized—*the advancement of medical science.* Select for the presiding officers those who, in deserving the position as a reward of distinguished labor, will reflect honor from their attainments, upon the character of the association.

"We trust that the amendment will have the effect ultimately to extinguish some of the *smaller lights* and give us an election that will be legitimate rather than accidental."

---

Mr. Paul A. Davis is our agent in Pennsylvania and New Jersey. Payments made to him will be duly acknowledged by us.

